# Shifts in Species Interactions Due to the Evolution of Functional Differences between Endemics and Non-Endemics: An Endemic Syndrome Hypothesis

**DOI:** 10.1371/journal.pone.0111190

**Published:** 2014-10-23

**Authors:** Courtney E. Gorman, Brad M. Potts, Jennifer A. Schweitzer, Joseph K. Bailey

**Affiliations:** 1 Dept. of Ecology and Evolutionary Biology, University of Tennessee, Knoxville, Tennessee, United States of America; 2 School of Plant Science, University of Tasmania, Hobart, Tasmania, Australia; University of New South Wales, Australia

## Abstract

Species ranges have been shifting since the Pleistocene, whereby fragmentation, isolation, and the subsequent reduction in gene flow have resulted in local adaptation of novel genotypes and the repeated evolution of endemic species. While there is a wide body of literature focused on understanding endemic species, very few studies empirically test whether or not the evolution of endemics results in unique function or ecological differences relative to their widespread congeners; in particular while controlling for environmental variation. Using a common garden composed of 15 *Eucalyptus* species within the subgenus *Symphyomyrtus* (9 endemic to Tasmania, 6 non-endemic), here we hypothesize and show that endemic species are functionally and ecologically different from non-endemics. Compared to non-endemics, endemic *Eucalyptus* species have a unique suite of functional plant traits that have extended effects on herbivores. We found that while endemics occupy many diverse habitats, they share similar functional traits potentially resulting in an endemic syndrome of traits. This study provides one of the first empirical datasets analyzing the functional differences between endemics and non-endemics in a common garden setting, and establishes a foundation for additional studies of endemic/non-endemic dynamics that will be essential for understanding global biodiversity in the midst of rapid species extinctions and range shifts as a consequence of global change.

## Introduction

Species ranges have been shifting since the Pleistocene [1], whereby fragmentation, isolation, and the subsequent reduction in gene flow have resulted in local adaptation of novel genotypes and the repeated evolution of endemic species. Endemic species have long been valued for their novelty by both the general and scientific communities, which has resulted in a vast body of evolutionary and natural history research [2]–[4]. However, in the midst of a biodiversity crisis where species extinction rates are 100 to 1000 times greater than the background geological rate [5], understanding the biology of endemic species has become a priority rather than a pursuit of novelty, as these species are often the ones most at risk [6]. Studies have investigated the causes and consequences of endemism [2]–[4], the geography, risks, and prospects of endemic species [7]–[10], as well as the genetic differences between endemic (or rare/narrowly distributed) vs. widespread species [11]–[13]. The literature is generally lacking, however, in studies that attempt to investigate the ecological significance of endemic species. Because the formation of relict populations and the evolution of endemic species is thought to be a major consequence of species range shifts due to climate change [14], identifying whether endemics are functionally different and support unique species interactions may place even greater conservation value on these populations and species.

Linking evolutionary history to contemporary ecological interactions is a burgeoning field that is bringing with it many new insights into the relationship between biodiversity, species interactions, and ecosystem function [15]. Despite studies on their evolutionary novelty, few studies have experimentally investigated the ecological differences between endemic species and their non-endemic congeners or how endemicity may influence species interactions; particularly while controlling for environmental variation. A 2003 study measured net photosynthesis, leaf nitrogen content, and specific leaf area of 78 crop, endemic, and non-endemic plant species [16]. They noted variation between endemic and non-endemic species, however did not find statistical significance for the observed differences between any of the measured parameters in the field. Additionally, a recent study compared traits of 20 congeneric pairs of endemic and widespread plant species and while they found that endemics were smaller and produced fewer flowers, they found no differences in traits related to resource acquisition, resource conservation, and patterns of herbivory [17]. While these studies provide a valuable basis for understanding the ecological differences between endemic and non-endemic species, they are limited in the inferences that they can make, since traits were measured *in situ* rather than in an experimental common garden and are thus influenced by a range of environmental variables. Common garden experiments provide an opportunity to more accurately partition genetic and environmental components of trait variation when attempting to characterize the ecology of a set of species [18] and provide a powerful tool for linking evolutionary history to contemporary ecological interactions.

Endemic species have frequently been characterized based on generalizations of their perceived commonalities, such as low genetic diversity [19], [11]–[13] and limited reproduction and dispersal abilities [20], [6]. For example, a 2000 study summarized the generalizations that are often made regarding the reproductive biology of endemic species as an increased tendency for self-compatibility, lower investment in reproduction, poorer dispersal abilities, and shorter generation times in comparison to common species [6]. Although attempts have been made to characterize endemic species based on their shared traits, the extent of this convergence on an endemic syndrome of traits remains unclear, along with how these shared differences may influence species interactions differently than those of common species. Here we hypothesize that endemic species are a homogenous group that can be characterized based on commonalities that result from isolation and lead to an ‘endemic syndrome’.

The genus *Eucalyptus* in Tasmania provides an ideal natural system for examining an endemic syndrome among congeners, as the island has 29 native eucalypts from two subgenera, 17 of which are endemic to the island of Tasmania, while the others also occur on the Australian mainland [21]. We used a common garden with 15 *Eucalyptus* species (9 endemic, 6 non-endemic) to test the hypothesis that functional plant traits and associated patterns of herbivory of endemic species differ from those traits in closely related non-endemic species. To our knowledge this is the first endemic/non-endemic comparative study to use an experimental common garden design to separate differential environmental conditions as explanatory variables. Here we show that endemic plant species are ecologically different than non-endemics. We show that these differences include functional plant traits with extended effects across trophic levels. Furthermore, we found that while endemics occupy many diverse habitats (from loamy sites near sea-level to alpine scrub), they share similar functional traits potentially resulting in an endemic syndrome of traits.

## Materials and Methods

### Common Garden

In order to test whether endemic species are ecologically different than non-endemics without the constraints of environmental/habitat variation, we used a common garden experiment. The common garden was part of a forestry trial established by The Cooperative Research Centre for Forestry (CRC). This experimental forest trial was established in 2009 with 15 species of closely related Eucalypts native to Tasmania that occur in the subgenus *Symphyomyrtus* [21]. Nine of these species are endemic to Tasmania, while the other 6 are native non-endemics that also occur on the Australian mainland. Both groups of species exhibit a widespread distribution within Tasmania and co-occur throughout the state. Non-endemic species included in the trial were *E. dalrympleana, E. rubida*, and *E. viminalis, E. brookeriana*, and *E. ovata* and *E. perriniana*. Endemic species included in the trial were *E. johnstonii*, *E. subcrenulata*, and *E. vernicosa*, *E. archeri, E. cordata, E. gunnii, E. morrisbyi*, and *E. barberi* and *E. rodwayi*. The endemic species included in the trial occupy a diverse variety of habitats ranging from loamy sites near sea-level (*E. cordata* and *E. morrisbyi*), poorly drained montane forest (*E. johnstonii*), well-drained subalpine rainforest (*E. subcrenulata*), and alpine scrub (*E. vernicosa*) [21]. Each species was represented by an average of four open-pollinated families collected from native trees in Tasmania with between 1 and 17 plants per family. Individuals were planted in rows that were 36 trees long. Plant positions within a row were allocated randomly, and the total sample size was 412 trees. Both mammalian and insect herbivores had unrestricted access to the garden. No specific permissions were required to carry out this study and field studies did not involve endangered or protected species.

### Plant Measurements

To quantify differences between endemics and their closely related non-endemic species, common plant functional traits (height, internode length, leaf thickness, and specific leaf area (SLA)) and herbivory were measured in 2011 on 4 year-old plants. Total tree height was measured to the nearest cm. Two random shoots and two fully expanded leaves were collected from the terminal stems of each tree (juvenile foliage) for measurements of shoot and leaf functional traits. Internode lengths (in mm) were measured on these shoots as the length between the first two fully expanded leaves; typically the 4^th^ and 5^th^ plastochron. Leaf thickness (in mm) was measured with digital calipers. Leaf length, width and area were estimated from the leaf samples using the imaging program ImageJ. Leaves were oven-dried at 70°C for 48 h. Specific leaf area (SLA) was calculated as the average leaf area/average dry weight (cm^2^/g).

To understand how potential functional differences between the endemic and non-endemic species might influence the response of interacting species, we quantified herbivory by common mammals and arthropods. Herbivory was estimated in three ways: total insect folivory on the whole tree, insect folivory on the most damaged branch, and total mammal browsing damage. Total insect folivory was visually surveyed and characterized as percent foliar tissue removed from 1–100 percent (i.e., 0, 1, 2, 3, 5, 10, 20, continuing in 10% increments). Because herbivory is often not uniform across an individual tree, a second survey was conducted on the most damaged branch of each tree using the same methodology. Characteristic shoot clipping by mammal browsing [22], typically by *Trichosurus vulpecula* (common brushtail possum) and *Thylogale billardierii* (red-bellied pademelon), was estimated on each tree as a total damage score. Scores were characterized as the percentage of shoot tips clipped from each tree (using the same scale as insect survey’s described above).

### Statistical Analyses

The data were analyzed using mixed effect models and Restricted Maximum Likelihood (REML) using the statistical program JMP 10. We tested for quantitative differences in several plant functional traits (height, internode length, leaf thickness, and specific leaf area (SLA)), as well as herbivory between endemic species and non-endemics. We used a conservative approach and constructed a mixed model that included seed family nested within tree species and row as random effects to account for variance explained by these factors that would otherwise contribute to differences between endemics and non-endemics. Endemism/non-endemism and tree species nested within endemism were included as fixed effects. Additionally, to account for multiple comparisons of traits between endemic and non-endemic species, we used the function ‘p.adjust’ in R (2.15.3) [23] to apply a Holm-Bonferroni correction to estimates of significance.

Because the divergence of *Eucalyptus* species in the subgenus *Symphyomyrtus* has been relatively recent, resolution of phylogenetic relationships at the species level has not been possible with standard DNA sequence markers [24]–[26]. This makes it impossible to use advanced comparative methods such as phylogenetically independent contrasts (PIC) to account for phylogenetic dependency of our data. In an attempt to account for phylogenetic dependency, we took a conservative approach and constructed a mixed model identical to the one above but that also included clade as a fixed effect. Clade was defined by taxonomic series (Ovatae or Viminales; [21]). Species included in the series Ovatae were *E. brookeriana, E. barberi, E. ovata,* and *E. rodwayi*, and species included in the series Viminales were *E. archeri, E. cordata, E. dalrympleana, E. gunnii, E. johnstonii, E. morrisbyi, E. perriniana, E. rubida, E. subcrenulata, E. vernicosa,* and *E. viminalis.*

Additionally, regression analyses were used to examine the relationships between plant functional traits (height, internode length, leaf thickness, and SLA) and percent foliar herbivory. Because variation in functional traits can reflect niche differentiation, we compared suites of functional traits that differed between endemic and non-endemic species using a two-dimensional ordination of multivariate data using Nonmetric Multidimensional Scaling (NMDS) (R 2.15.3, vegan package) [27]. A distance matrix was constructed using Euclidean distances based on the values of functional plant traits and patterns of herbivory, which were standardized by maximum resemblance for all individuals in the study. Differences were quantified using ANOSIM (analysis of similarity) (R 2.15.3, vegan package) [27], a non-parametric method for determining if there is significant variation between groups of samples based on a Euclidean distance [28].

## Results

### Functional trait differences between endemic and non-endemic species

**Figure 1:**
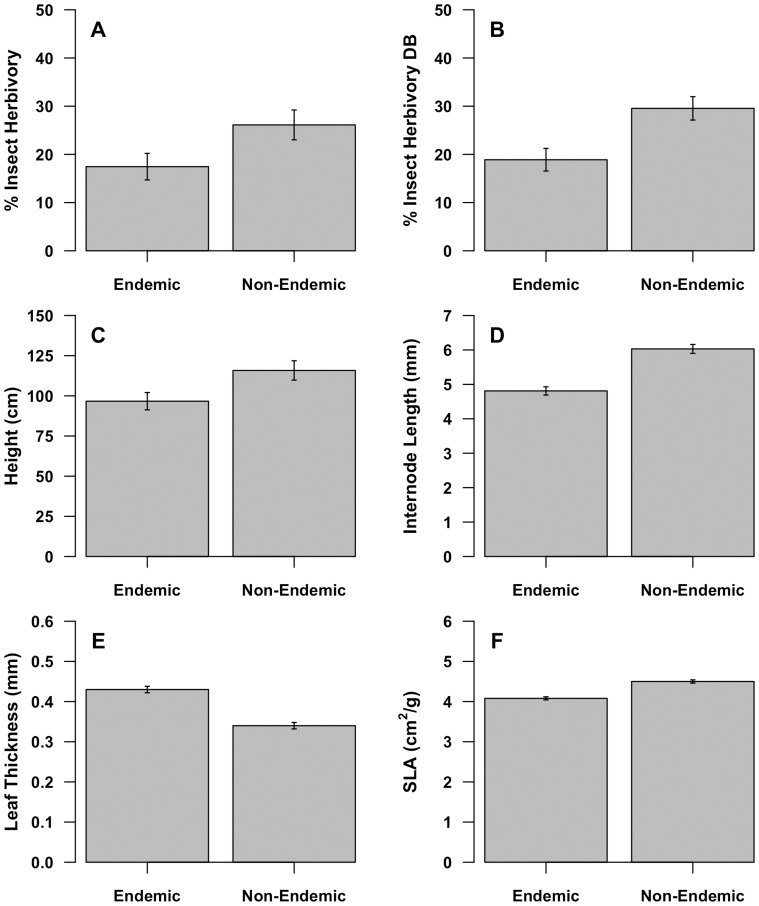
Functional traits and patterns of herbivory differ between endemic *Eucalyptus* species and their non-endemic congeners. Variation in plant functional traits and insect herbivory relative to level of endemism: (A) total foliar herbivory (B) foliar herbivory on the most damaged branch (DB), (C) height, (D) internode length, (E) leaf thickness, (F) specific leaf area (SLA). Total sample size was 412 trees. Error bars represent the standard error.

**Figure 2:**
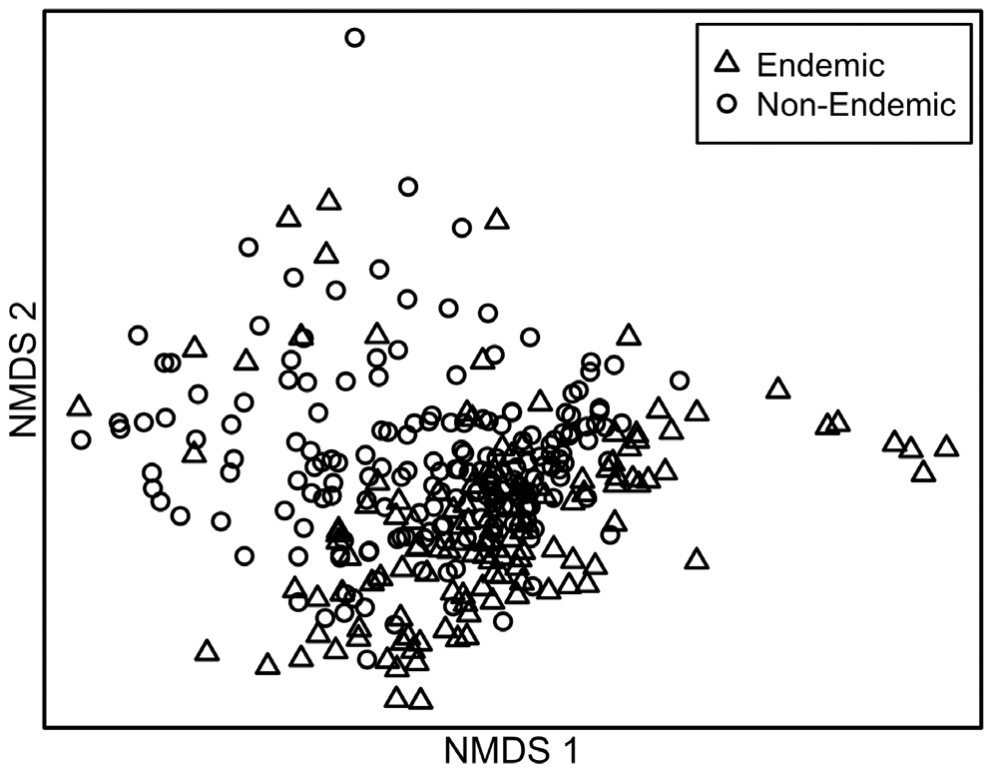
Endemic *Eucalyptus* species contribute a unique suite of functional traits to the landscape. Non-metric multidimensional scaling (NMDS) plot using functional trait and herbivory data showing separation between suites of functional traits and patterns of herbivory of endemic species versus non-endemics. Open circle symbols represent non-endemic species and open triangle symbols represent endemic species.

The endemic species differed from non-endemic species in functional plant traits. Height, internode length, leaf thickness, and specific leaf area varied significantly between endemic and non-endemic *Eucalyptus* species (**Table 1**, **Figure 1**). The endemic species had 22% thicker leaves (Figure 1E) and 9% lower average SLA (Figure 1F) than the non-endemic congeners. The endemics also had 23% shorter internodes (Figure 1D) and were 18% shorter in height (Figure 1C) than their non-endemic congeners (Table 1). The Holm corrected estimates of significance generally supported our inferences from uncorrected p-values (Table 1). When the functional traits were combined in a multivariate framework, there were significant differences between the endemic and non-endemic species (**Figure 2**, ANOSIM: R = 0.119, p<0.001) providing evidence of an endemic syndrome of traits.

**Table 1:** Mixed model analysis of plant functional traits.

Response Variable	F_1,13_	p
% Insect Herbivory	4.446	0.039* (0.078)
% Insect Herbivory DB	9.932	0.002* (0.008*)
% Mammal Herbivory	0.454	0.502 (0.502)
Specific Leaf Area (cm^2^/g)	41.919	<0.001* (0.001*)
Height (cm)	5.588	0.021* (0.063)
Internode Length (mm)	45.066	<0.001* (0.001*)
Leaf Thickness (mm)	49.318	<0.001* (0.001*)

Summary of mixed model analysis using Restricted Maximum Likelihood (REML) of the differences between samples of endemic (*n* = 9) and non-endemic (*n* = 6) eucalypt species of the subgenus *Symphyomyrtus* growing on the island of Tasmania. Holm- Bonferroni corrected *p*-values are given in parentheses.

Endemic species also exhibited less herbivory than non-endemic species. The endemic species had 40% less total insect folivory (Figure 1A) and 44% less herbivory on the most damaged branch (Figure 1B) than the non-endemics (Table 1). Additionally, the response of insect herbivores was correlated with plant functional traits (height, internode length, leaf thickness, and specific leaf area) (**Table 2**). Significant differences in mammal browsing were not detected (p = 0.502); all trees experienced ∼10.5% of mammal damage.

**Table 2:** Multiple regression model results for leaf traits on herbivore response.

	Coefficient	Standard Error	*p*	R^2^
Height (cm)	0.211	2.779	<0.001*	0.152
Internode Length (mm)	4.195	1.041	<0.001*	0.048
Leaf Thickness (mm)	−62.654	13.018	<0.001*	0.067
SLA (cm^2^/g)	7.610	3.222	0.018*	0.017

Summary of regression analysis of the correlation between plant functional traits and total foliar herbivory (*n* = 412).

It is also possible that shared evolutionary history could influence the differences between endemic and non-endemic species in functional traits. When evolutionary history was accounted for in the mixed model, the levels of significance of endemism as a fixed effect did not change among internode length, leaf thickness, and specific leaf area (**Table 3**), suggesting that shared evolutionary history was not driving the differences in functional traits or patterns of herbivory.

**Table 3:** Mixed model analysis of functional trait measures including clade as a fixed effect.

	Endemism		Clade	
Response Variable	F_1,13_	p	F_1,13_	p
% Insect Herbivory	1.572	0.232	0.987	0.337
% Insect Herbivory DB	2.768	0.121	0.016	0.901
Height (cm)	1.518	0.248	0.741	0.411
Internode Length (mm)	5.671	0.038*	0.065	0.805
Leaf Thickness (mm)	7.633	0.017*	0.904	0.361
Specific Leaf Area (cm^2^/g)	11.794	0.005*	1.267	0.282

Summary of mixed model analysis using Restricted Maximum Likelihood (REML) for the difference between samples of endemic (*n* = 9) and non-endemic (*n* = 6) eucalypt species of the subgenus *Symphyomyrtus* growing on the island of Tasmania when evolutionary history is accounted for.

## Discussion

This study demonstrates that despite having evolved from sea-level to tree-line, and under a broad range of selective pressures, endemic *Eucalyptus* species are functionally different from closely related non-endemic congeners. These results support a general hypothesis of convergence on an endemic syndrome of traits. Specifically, we found that endemics have more stress tolerant resource acquisition traits, such as lower SLA, thicker leaves, shorter internodes, and slower growth than widespread, non-endemic species. Although studies involving more species are required to fully understand the driving forces behind these differences, we believe that convergent evolution in response to an environmental gradient (such as elevation or harsh soil conditions) is likely playing a substantial role in the differences in functional traits that we found. Regardless of the environmental conditions driving this convergence, such functional differences in plant traits between endemics and non-endemics reflect differences in nutritional quality and palatability of these species, which in turn likely impacted the response of insect herbivores.

In general, the functional plant traits associated with the endemic species reflect a poorer quality resource for herbivores. For example, we found endemics to have lower SLA than non-endemics, a trait correlated with water use, leaf life span, and leaf nitrogen content [29]. We also found that the endemic species experienced less insect herbivory than non-endemics. This is consistent with the resource availability hypothesis [30] that suggests that the local environment heavily influences anti-herbivore defenses, and that plants with traits such as slow growth rates and long leaf lifespans generally invest more in anti-herbivore defense. Additionally, the response of herbivores was correlated with internode length, leaf thickness, and SLA. While this result suggests that endemics represent a poorer quality resource for herbivores than non-endemics, the alternate hypothesis, that the herbivores specialized for the endemic or non-endemic species were absent from the common garden, cannot be dismissed. Additionally, significant differences in mammal browsing were not detected. However, this result is inconsistent with those from a 2002 study of eucalypt susceptibility to marsupial damage that found that the endemic species *E. gunnii* and *E. morrisbyi* are significantly more susceptible to possum browsing than the two non-endemic species used in the study (*E. globulus* and *E. ovata*) [31]. More studies should examine both insect and mammalian herbivory to determine if there are general differences between endemic and non-endemic species in this ecologically important interaction.

Endemic species are highly valued from a biodiversity standpoint, as the scientific community has made preventing extinctions an urgent priority [32]. Our research shows that endemic *Eucalyptus* species contribute a novel syndrome of traits, with extended consequences across trophic levels (i.e., endemic species experienced less herbivore damage). These results contribute to a growing body of research that suggests genetically based plant traits can have direct and indirect effects on communities [33]–[35], that can in turn influence ecosystem processes [36]–[37]. For example, a recent study showed that variation in species interactions has major consequences for community composition and ecosystem processes, such as energy flow, that increase across levels of organization [37]. This has important implications for the conservation of biodiversity, as the loss of endemics as a group might also represents the loss of novel ecological interactions.

Endemic plant species generally evolve in response to a broad range of environmental conditions, including edaphic factors, altitude, geographic isolation, and several other ecological conditions. In the context of climate change where species ranges have been shifting since the Pleistocene [1], fragmentation, isolation, and the subsequent reduction in gene flow have resulted in local adaptation of novel genotypes and the evolution of endemics [38]–[41], [14]. It remains to be seen if endemics across gradients are commonly different from closely related non-endemics, but our results provide a testable hypothesis for endemic syndromes that is worthy of future attention across plant systems. Much more research is needed to elucidate the causes and consequences of the evolution of endemism and to understand whether the conservation of endemics also preserves a unique suite of species interactions.
